# Acquired Thrombotic Thrombocytopenic Thrombocytopenia Presenting As Urinary Tract Infection and Perianal Abscess: A Case Review

**DOI:** 10.7759/cureus.57950

**Published:** 2024-04-10

**Authors:** Oluwaremilekun Tolu-Akinnawo, Kikelola Oyeleye, Ikenna Nnamani

**Affiliations:** 1 Internal Medicine, Meharry Medical College, Nashville, USA

**Keywords:** thrombocytopenia, microangiopathic autoimmune hemolytic anemia, hemolytic anemia, adamst13 deficiency, perianal abscess, acquired ttp, uti

## Abstract

Thrombotic thrombocytopenic purpura (TTP) is a rare but potentially life-threatening hematologic disorder characterized by hemolytic anemia, thrombocytopenia, renal failure, fever, and neurologic dysfunction. While cases often do not present with all five characteristics (<5%), TTP can be hereditary or acquired, often due to a deficiency or dysfunction of the ADAMST13 enzyme. Here, we describe a case of infection-induced acquired TTP in a middle-aged male with urinary tract infection (UTI) and perianal abscess. Suspicion arose from hematologic abnormalities, fever, thrombocytopenia, acute renal failure, and the presence of an underlying infection. A PLASMIC score of 6 (indicating a 72% probability of ADAMTS13 deficiency) prompted ADAMTS13 level testing, revealing levels <5% with the presence of an inhibitor, confirming TTP diagnosis. Treatment with high-dose steroids and daily plasma exchange yielded a swift platelet response, necessitating only two to three days of plasma exchange. In addition, incision and drainage of the perianal abscess were performed. The patient was discharged on daily prednisone and initiated on four doses of weekly Rituximab to mitigate recurrence risk. This case underscores the importance of early suspicion and treatment in infectious triggers such as UTI/perianal abscess, offering crucial diagnostic and prognostic insights.

## Introduction

Thrombotic thrombocytopenic thrombocytopenia is an infrequent hematologic disorder that can be potentially fatal if not diagnosed and adequately treated early [1]. It can be inherited or acquired, just as in the case of our patient. An estimated three cases per one million adults per year [2]. It is more common in adults compared to the closely related hematologic disorder, a hemolytic uremic syndrome more common in children [1]. Both are characterized by thrombocytopenia and microangiopathic hemolytic anemia; however, neurologic abnormalities are characteristic of thrombotic thrombocytopenic thrombocytopenia [1]. Thrombotic thrombocytopenic thrombocytopenia is caused by severely reduced activity of von Willebrand factor (VWF) - cleaving protease - ADAMTS13. The diagnosis is based on the presence of pentad thrombocytopenia, microangiopathic hemolytic anemia, neurologic abnormalities, and fever (although pentad rare); however, suspicion should not delay management due to the fatality of late treatment [1].

The etiology of thrombotic thrombocytopenic thrombocytopenia remains unclear; however, it has been linked to various conditions such as sepsis, autoimmune disorders, malignancies, and pregnancies [1]. We report a case of thrombotic thrombocytopenic thrombocytopenia in a patient with urinary tract infection (UTI) and perianal abscess, which appears to be rare, the clues to diagnosis, treatment, and the post-treatment outcome.

## Case presentation

This case involves a 62-year-old male with a medical history including cerebrovascular accident (CVA), hyperlipidemia, hypertension, thrombosed hemorrhoids (status-post excision two years before the current presentation), and constipation. The patient, who had not been compliant with any of his medications, presented to the emergency room (ER) with a one-week history of left-sided perianal pain. His vital signs were a temperature of 98 °F, blood pressure of 150/102 mmHg, pulse rate of 92 beats per minute, respiratory rate of 18 breaths per minute, and oxygen saturation of 98% on room air. On physical exam, his abdomen was soft, nontender, and nondistended, with no hepatosplenomegaly and normoactive bowel sounds in all four quadrants. A rectal exam revealed brown stool around the perianal region, normal sensation, and rectal tone, as well as tenderness upon palpation, which was most pronounced at the 9 o'clock position. Additionally, a small lump approximately 1 x 1 cm in size was palpated at the same 9 o'clock position. The rectal examination was limited by perianal pain.

**Figure 1:**
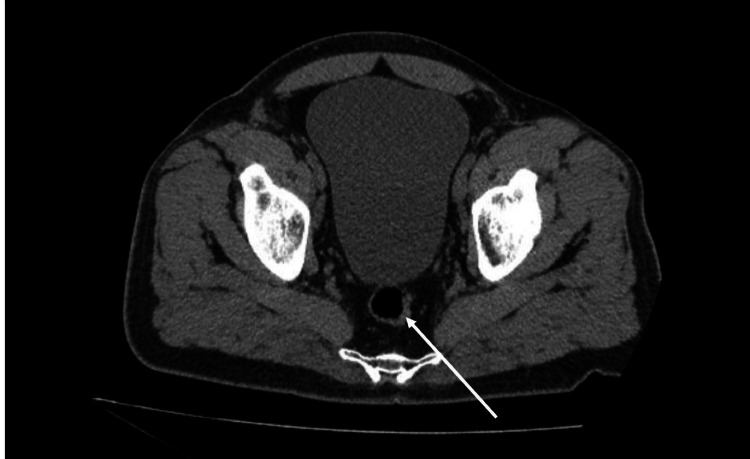
CT scan of the abdomen and pelvis showing the perianal abscess (white arrow). CT, computed tomography

A complete blood count (CBC) revealed a normal white cell count (WBC), anemia, and thrombocytopenia, as illustrated in Table 1. There was also a new acute kidney injury (AKI) with creatinine of 1.92 mg/dL versus 1.25 mg/dL the previous month. Urinalysis showed 1+ glucose (reference negative), 452 red blood cells (reference 0-5), 3+ blood (reference negative), 15 white blood cells (reference 0-5), and negative leukocyte esterase. Abdominal CT scan revealed extensive inflammatory changes surrounding both kidneys without significant hydronephrosis, nonobstructive calculi in the left kidney, moderately distended bladder, and a 20 mm x 27 mm soft tissue swelling/fullness in the left perirectal region that may represent a small perianal abscess (Figure 1).

**Table 1:** Laboratory trend of our patient.

Labs	Day 1	Day 2	Day 3	One week post-discharge	Reference
White cell count (WBC) (x10^3^/µL)	10.2	10.1	9.3	8.9	4.5-11.0
Hemoglobin (HBG) (g/dL)	13.4	12.5	9.9	9.5	13.5-17.5
Platelet count (x10^3^/µL)	42	21	9	282	150-400
Creatinine (mg/dL)	1.92	2.5	3.2	1.5	0.6-1.2

The patient was started on piperacillin/tazobactam for the perianal abscess and possible pyelonephritis. General surgery was consulted, and conservative management was recommended, given the abscess size. Thrombocytopenia worsened on repeat CBC, as seen in Table 1. Renal function also declined, with worsening AKI. Zosyn was changed to ampicillin/sulbactam due to concern for possible contribution to thrombocytopenia.

Further workup revealed a blood smear with schistocytes, repeat total bilirubin of 1.4 mg/dL (reference 0-2.0 mg/dL), lactate dehydrogenase (LDH) elevated at 998 U/L (reference 140-280 U/L), haptoglobin undetectable (reference 0.33-3.46 g/L), and reticulocytes inappropriately low at 1.22% (reference 0.5%-2%, however, expected to be increased in hemolysis). The fractional excretion of sodium (FeNa) was calculated to be 1.2% (reference <0.1%). Renal ultrasound showed significant postvoid residual urine within the bladder, and mildly increased echogenicity of the kidney without hydronephrosis may suggest an element of medical renal disease. Nephrology and hematology/oncology were consulted for further recommendations.

His platelet count decreased further to 9, and the patient received a platelet transfusion. Hemoglobin continued to trend down, with AKI worsening. The Coombs test was negative. Initial prothrombin and partial thromboplastin times were normal at 14.2 and 36.6 seconds, respectively. Repeat testing two days later showed a slightly prolonged prothrombin time at 15.5 (reference <15) seconds and a partial thromboplastin time of 40.4 (reference < 35) seconds. The international normalized ratio (INR) was 1.2 (reference 0.9-1.2), while the mean corpuscular volume (MCV) was 76 (reference 80-100). Of note, all of the patient's laboratory results were relatively normal before the current presentation. Table 1 provides a graphical representation of the laboratory findings.

Due to the absence of reticulocytosis, a plan was made for a possible bone marrow biopsy at a later date. The most likely differential diagnoses were considered to be thrombotic thrombocytopenic purpura (TTP) and atypical hemolytic uremic syndrome (HUS). He was started on fresh frozen plasma twice a day. A PLASMIC score was calculated to be 6 (low platelets, MCV < 90fL, INR < 1.5, and haptoglobin undetectable), suggestive of a 72% probability of ADAMTS13 deficiency. ADAMTS13 levels were obtained and found to be reduced to < 5%, along with the presence of an inhibitor, confirming the diagnosis of TTP. He began prednisone at a dosage of 1 mg/kg/day and pantoprazole, with plans to initiate plasma exchange. Subsequently, he was transferred to an outside hospital due to a lack of hematology coverage. He was treated with high-dose steroids and 1.5x plasma volume exchange. His platelet response was brisk, requiring only two to three days of plasma exchange (PLEX). He also underwent an incision and drainage of the perianal abscess. He was discharged home on tapered daily prednisone. He returned for an outpatient follow-up at the hematology clinic. He started on four doses of weekly rituximab to decrease the risk of recurrence.

## Discussion

Thrombotic thrombocytopenic thrombocytopenia is a microangiopathic hemolytic anemia syndrome accompanied by thrombocytopenia, renal insufficiency, fever, and neurological disorders [3]. Although it can be inherited, acquired thrombotic thrombocytopenic thrombocytopenia is primarily idiopathic and may be attributed to various inflammatory conditions (e.g., hepatitis C, pneumonia, HIV, and sepsis), medications such as mitomycin C, and chronic diseases such as malignancies [3].

Acute pancreatitis has also been reported in the literature, responsible for about 2% of cases [2,4]. Although bacterial infections and sepsis have been identified as triggers for thrombotic thrombocytopenic thrombocytopenia, there have not been any reported cases of perianal abscess. However, a hepatic abscess was reported due to Actinomyces turicensis [5]. However, the association between Thrombotic thrombocytopenic purpura and UTI remains debated, with at least two cases reported in the literature [6,7].

The pathophysiology of thrombotic thrombocytopenic thrombocytopenia is related to inherited or acquired deficiency or dysfunction of ADAMTS13, which subsequently leads to the accumulation of large polymers of VWF molecules causing platelet-rich microvascular thrombosis [8]. VWF responsible for linking platelets and the blood vessel wall for hemostasis is usually broken down by ADAMTS13. During deficiency or dysfunction of ADAMTS13, there is an increased accumulation of VWF, leading to an increased propensity for coagulation. The large VWF proteins promote activation and aggregation, forming thrombi [9]. Continuous formation of thrombi leads to increased platelet consumption, thereby leading to a reduced platelet count. The formation of thrombi also leads to end-organ damage due to vessel occlusion. Hemolytic anemia results from the fragmentation of red blood cells as they pass through the thrombi-occluded vessels, giving a characteristic appearance called schistocytes. Organ damage is primarily in the brain (leading to neurological symptoms) and kidneys (leading to renal failure). The clinical presentation of patients with thrombotic thrombocytopenic thrombocytopenia varies depending on the etiology. In the case of our patient, fever, and complications from anemia were the predominant features. Laboratory investigations are crucial to diagnosis. A low hematocrit with an elevated reticulocyte count, elevated indirect bilirubin level, and decreased haptoglobin level indicates hemolytic anemia. High LDH levels indicate ongoing hemolysis as well as possible end-organ damage and can be used to monitor the disease's progression [10,11]. Elevated creatinine indicates renal dysfunction, as seen in our patient. Low platelets are also very typical, as seen in our patients. Coagulation studies are, however, usually normal. When suspicion is high, a low ADAMTS13 (<10%) level is often diagnostic of thrombotic thrombocytopenic thrombocytopenia. However, therapy is initiated based on clinical diagnosis.

The association between thrombotic thrombocytopenic thrombocytopenia and UTI continues to be up for debate. Molecular mimicry between antibodies directed against the infectious agent and the targeting ADAMTS13 has been suggested as a possible cause [7]. A recent retrospective study that examined the incidence of UTIs in patients with idiopathic thrombocytopenic purpura between 1999 and 2007 identified a significant incidence of UTIs in patients with TTP, consistent with current literature [7].

Several hypotheses have been proposed regarding possible associations. First, there is a potential molecular mimicry between antibodies directed against infectious agents and the targeting ADAMTS13 protease. Second, the underlying infection (such as UTI or perianal abscess in the case of our patient) causes endothelial damage that increases the formation of microthrombi. Studies have also shown that patients with severe infections (e.g., UTI and perianal abscess as in the case of our patient) have a higher level of VWF antigen compared with healthy control subjects and patients without sepsis [12]. Also, the release of ultra-large VWF can be stimulated by inflammatory cytokines such as interleukin-8 and tumor necrosis factor, with interleukin-6 inhibiting the cleavage of ultra-large VWF [13]. This subsequently promotes coagulation and thrombi formation.

Before the introduction of therapeutic plasma exchange to treat TTP, the fatality rate was as high as 90% [14]. Medications are associated with <15% of all cases of TTP and need to be identified and stopped [15]. However, none of our patients' medications have been implicated in developing TTP, and he was currently not compliant with any of his medications at the time of presentation. The main stem of treatment of TTP remains plasma exchange [16,17]. If plasma exchange is unavailable, patients should be treated with fresh frozen plasma until plasma exchange becomes available, as was the case with our patient [18]. In addition to immunosuppressive agents, including steroids, a novel agent called Caplacizumab (Cablivi), which works by binding to the A1 domain of VWF, thereby blocking the aggregation of platelets, has been approved for the treatment of TTP in adults [19]. Splenectomy, vincristine, cyclophosphamide, azathioprine, and high-dose immunoglobulins have also been used in refractory cases. Tapering of plasma exchange for two to three days has also been recommended to prevent relapses, which can be as high as 30%-60%, once the platelet count is normal. Newer recommendations include chimeric monoclonal anti-CD20, which decreases the level of autoantibodies and is an effective treatment for TTP [20-24]. This was utilized in the case of our patient.

The mortality rate of TTP is greater than 90% for untreated patients; however, early treatment with plasma exchange decreases this risk to 10%-20% [25-28]. Our patient benefitted from early diagnosis and treatment, highlighting the importance of awareness among healthcare providers.

## Conclusions

This case illustrates the connection between TTP and infectious triggers, specifically UTIs and perianal abscesses. It highlights the ongoing debate regarding the exact relationship between TTP and UTIs while emphasizing the critical need for vigilance in identifying TTP in septic patients. Early diagnosis and treatment initiation are deemed paramount to prevent severe consequences. Additionally, it advocates for further investigation into the genetic predisposition to acquired TTP, suggesting that understanding genetic determinants could lead to more targeted therapeutic interventions and enhance patient management. In summary, the conclusion stresses the importance of considering TTP as a potential complication in septic patients and calls for continued research efforts to advance our understanding of the disorder and improve patient outcomes.

## References

[1] Bhandari A, Pokhrel B, Oli PR, Le Q, Basnet B, Freitag EC, Nayani A (2023). A rare case of thrombotic thrombocytopenic purpura (TTP) with concurrent renal cell carcinoma: diagnostic and therapeutic challenges. Cureus.

[2] Sravanthi MV, Suma Kumaran S, Sharma N, Bojanapally P (2023). A rare case of acquired thrombotic thrombocytopenic purpura triggered by acute pancreatitis. Cureus.

[3] Rawala MS, Naqvi ST, Khan MY, El Toukhy A (2023). A rare case of thrombotic thrombocytopenic purpura caused by pancreatitis and clopidogrel. Am J Case Rep.

[4] Wang CH, Jin HF, Liu WG, Guo Y, Liu Z (2022). Acute pancreatitis-induced thrombotic thrombocytopenic purpura: a case report. World J Clin Cases.

[5] Riegert-Johnson DL, Sandhu N, Rajkumar SV, Patel R (2002). Thrombotic thrombocytopenic purpura associated with a hepatic abscess due to Actinomyces turicensis. Clin Infect Dis.

[6] Ardalan M (2014). Urinary tract infection associated with thrombotic microangiopathy. Nephrourol Mon.

[7] Park YA, Schultz EF, Hay SN, Brecher ME (2011). Thrombotic thrombocytopenic purpura and urinary tract infections: is there a connection?. Am J Clin Pathol.

[8] McDonald V, Laffan M, Benjamin S, Bevan D, Machin S, Scully MA (2009). Thrombotic thrombocytopenic purpura precipitated by acute pancreatitis: a report of seven cases from a regional UK TTP registry. Br J Haematol.

[9] Asada Y, Sumiyoshi A, Hayashi T, Suzumiya J, Kaketani K (2023). Immunohistochemistry of vascular lesion in thrombotic thrombocytopenic purpura, with special reference to factor VIII related antigen. Thromb Res.

[10] Thompson CE, Damon LE, Ries CA (1992). Thrombotic microangiopathies in the 1980s: clinical features, response to treatment, and the impact of the human immunodeficiency virus epidemic. Blood.

[11] Cohen JA, Brecher ME, Bandarenko N (1998). Cellular source of serum lactate dehydrogenase elevation in patients with thrombotic thrombocytopenic purpura. J Clin Apher.

[12] Kayal S, Jaïs JP, Aguini N, Chaudière J, Labrousse J (1998). Elevated circulating E-selectin, intercellular adhesion molecule 1, and von Willebrand factor in patients with severe infection. Am J Respir Crit Care Med.

[13] Bernardo A, Ball C, Nolasco L, Moake JF, Dong JF (2023). Effects of inflammatory cytokines on the release and cleavage of the endothelial cell-derived ultralarge von Willebrand factor multimers under flow. Blood.

[14] Rock GA, Shumak KH, Buskard NA, Blanchette VS, Kelton JG, Nair RC, Spasoff RA (1991). Comparison of plasma exchange with plasma infusion in the treatment of thrombotic thrombocytopenic purpura. Canadian Apheresis Study Group. N Engl J Med.

[15] Scully M, Hunt BJ, Benjamin S (2012). Guidelines on the diagnosis and management of thrombotic thrombocytopenic purpura and other thrombotic microangiopathies. Br J Haematol.

[16] Apte SS (2004). A disintegrin-like and metalloprotease (reprolysin type) with thrombospondin type 1 motifs: the ADAMTS family. Int J Biochem Cell Biol.

[17] Bukowski RM, King JW, Hewlett JS (1977). Plasmapheresis in the treatment of thrombotic thrombocytopenic purpura. Blood.

[18] Byrnes JJ, Lian EC (1979). Recent therapeutic advances in thrombotic thrombocytopenic purpura. Semin Thromb Hemost.

[19] Katsivalis KV, Thomas J (2021). Caplacizumab for acute thrombotic thrombocytopenic purpura. J Adv Pract Oncol.

[20] Yomtovian R, Niklinski W, Silver B, Sarode R, Tsai HM (2004). Rituximab for chronic recurring thrombotic thrombocytopenic purpura: a case report and review of the literature. Br J Haematol.

[21] Gutterman LA, Kloster B, Tsai HM (2002). Rituximab therapy for refractory thrombotic thrombocytopenic purpura. Blood Cells Mol Dis.

[22] Fakhouri F, Vernant JP, Veyradier A (2005). Efficiency of curative and prophylactic treatment with rituximab in ADAMTS13-deficient thrombotic thrombocytopenic purpura: a study of 11 cases. Blood.

[23] Scully M, Cohen H, Cavenagh J (2007). Remission in acute refractory and relapsing thrombotic thrombocytopenic purpura following rituximab is associated with a reduction in IgG antibodies to ADAMTS-13. Br J Haematol.

[24] Schleinitz N, Ebbo M, Mazodier K (2007). Rituximab as preventive therapy of a clinical relapse in TTP with ADAMTS13 inhibitor. Am J Hematol.

[25] Vesely SK, George JN, Lämmle B, Studt JD, Alberio L, El-Harake MA, Raskob GE (2023). ADAMTS13 activity in thrombotic thrombocytopenic purpura-hemolytic uremic syndrome: relation to presenting features and clinical outcomes in a prospective cohort of 142 patients. Blood.

[26] Raife T, Atkinson B, Montgomery R, Vesely S, Friedman K (2023). Severe deficiency of VWF-cleaving protease (ADAMTS13) activity defines a distinct population of thrombotic microangiopathy patients. Transfusion.

[27] Zheng XL, Kaufman RM, Goodnough LT, Sadler JE (2004). Effect of plasma exchange on plasma ADAMTS13 metalloprotease activity, inhibitor level, and clinical outcome in patients with idiopathic and nonidiopathic thrombotic thrombocytopenic purpura. Blood.

[28] Coppo P, Bengoufa D, Veyradier A (2004). Severe ADAMTS13 deficiency in adult idiopathic thrombotic microangiopathies defines a subset of patients characterized by various autoimmune manifestations, lower platelet count, and mild renal involvement. Medicine (Baltimore).

